# A Randomized Trial of Pharmacogenetic Warfarin Dosing in Naïve Patients with Non-Valvular Atrial Fibrillation

**DOI:** 10.1371/journal.pone.0145318

**Published:** 2015-12-28

**Authors:** Vittorio Pengo, Carlo-Federico Zambon, Paola Fogar, Andrea Padoan, Giovanni Nante, Michela Pelloso, Stefania Moz, Anna Chiara Frigo, Francesca Groppa, Dania Bozzato, Enrico Tiso, Elisa Gnatta, Gentian Denas, Seena Padayattil Jose, Roberto Padrini, Daniela Basso, Mario Plebani

**Affiliations:** 1 Department of Cardiac, Thoracic, and Vascular Sciences University of Padova, Padova, Italy; 2 Department of Medicine-DIMED, University of Padova, Padova, Italy; 3 Department of Laboratory Medicine, University of Padova, Padova, Italy; University of Perugia, ITALY

## Abstract

Genotype-guided warfarin dosing have been proposed to improve patient’s management. This study is aimed to determine whether a *CYP2C9- VKORC1- CYP4F2-*based pharmacogenetic algorithm is superior to a standard, clinically adopted, pharmacodynamic method. Two-hundred naïve patients with non-valvular atrial fibrillation were randomized to trial arms and 180 completed the study. No significant differences were found in the number of out-of-range INRs (INR<2.0 or >3.0) (p = 0.79) and in the mean percentage of time spent in the therapeutic range (TTR) after 19 days in the pharmacogenetic (51.9%) and in the control arm (53.2%, p = 0.71). The percentage of time spent at INR>4.0 was significantly lower in the pharmacogenetic (0.7%) than in the control arm (1.8%) (p = 0.02). Genotype-guided warfarin dosing is not superior in overall anticoagulation control when compared to accurate clinical standard of care.

**Trial Registration:**

ClinicalTrials.gov NCT01178034

## Introduction

Despite the introduction of new oral anticoagulants with a more predictable dose response and no need for laboratory monitoring, warfarin remains the most commonly prescribed oral anticoagulant worldwide. The Achilles’ heel of warfarin use is, nevertheless, the drug’s wide inter-individual variability in dose requirements. This makes it difficult to identify optimal loading/maintenance doses and leads to hemorrhagic events particularly during the initial treatment period [1]. Several methods have been proposed to safely initiate warfarin, and researchers’ efforts were intensified when specific gene polymorphisms, affecting warfarin pharmacokinetics or pharmacodynamics, were identified [2–5].

A number of genetic-based algorithms have been developed and proposed to guide clinicians in predicting optimal warfarin maintenance doses in their patients [6]. Two of these, which were validated by large population studies [7,8], are freely available on-line and some even calculate a loading dose to quickly attain stable warfarin plasma levels [9,10].^-^ Only a few prospective controlled trials have, nevertheless, been carried out to assess if these personalized approaches are superior—in terms of international normalized ratio (INR) control and prevention of major bleedings or thromboembolic events—to traditional/standard “trial-and-error” dosing methods [11–17]. Among them, two recently published randomized trials compared the genotype-guided warfarin dosing with standard or clinically-guided dosing and reported conflicting results [15–16].

We previously developed and validated a pharmacogenetic algorithm based on the demographic and genetic characteristics of Caucasian population [18]. Aim of the present study was to compare this algorithm with the pharmacodynamic approach currently used in our Institution [19].

## Materials and Methods

### Study design

This is a single centre, single-blinded, randomized study aimed at the early identification of optimal approach of warfarin dosing in naïve patients. Consecutive patients with non-valvular atrial fibrillation, aged >18 years and referred to local Thrombosis Centre to initiate oral anticoagulant treatment with warfarin were considered eligible for the study (S1 File). The patients who: were or might become pregnant, were receiving medication with amiodarone or heavy CYP-450 inducers (rifampin and carbamazepine), had a baseline INR>1.2 were excluded from the study (S1 File). Patients were allocated at enrolment to one of the study arms by blocked randomization, using randomly varying block sizes of 2, 4 and 6 subjects. Patients were blinded to the arm to which they had been assigned. All participants provided written informed consent. The study complies with the Helsinki Declaration, was approved by the Ethics Committee of the University Hospital of Padova on April 14^th^ 2008 (record number 1643P). The first patient was recruited on October 1^st^ 2009 and the follow up lasted on October 17^th^ 2012. The trial was registered on August 6^th^ 2010 at the U.S. National Institutes of Health (ClinicalTrials.gov Identifier: NCT01178034, available at https://clinicaltrials.gov/ct2/show/NCT01178034?term=warfarin+padova&rank=1) after patient recruitment began when we recognized the importance of registering the trial at an international registry although it was a single centre national study. The authors confirm that all ongoing and related trials for this drug/intervention are registered.

### Patients, data collection, and warfarin dosing

Physicians of the local Thrombosis Centre enrolled patients on Monday, Tuesday and Wednesday. Peripheral blood samples were collected, consecutively numbered and sent to the Laboratory for the baseline INR measurement (ACL Top 500 Instrumentation Laboratory, with RecombiPlasTin 2G Instrumentation Laboratory, Milano Italy) and DNA extraction. Laboratory personnel involved in the study allocated patients on the basis of the randomization sequence generated by statistician using Stata version 12 (StataCorp, TX, USA) for Windows. Genotyping for *CYP2C9*, *VKORC1* and *CYP4F2* was performed on Wednesday as previously described [18] and further presented in S2 File. Patients were prescribed enoxaparin (4000 IU q.d. subcutaneously) from day of enrolment un till warfarin initiation (Thursday, day 1 of treatment). INR was checked on days 5, 7, 9, 12, 15, 19 of treatment and subsequently according to the attending physician for a minimum follow up period of 30 days.

In the control arm warfarin 5mg was administered daily for the first 4 days (day 1 –day 4); day 5 and day 6 dosing were derived from the pharmacodynamic prediction model based on day 5 INR result as previously described [19] and further presented in S3 File. In the pharmacogenetic arm the personalized loading dose administered on day 1 and the subsequent maintenance dose (from day 2 to day 6) were calculated using the pharmacogenetic algorithm previously described [18] and further detailed in S4 File. The maximum loading dose was set at 10mg as a safety precaution. Starting on day 7, warfarin dosing was determined in both study arms by the attending physician with the assistance of PARMA v5.7 software [20].

### Outcomes

The primary study outcome measures, evaluated over the first 19 days of warfarin treatment, were the number of out-of-range INRs (INR<2.0 or >3.0), and the percentage of time spent in the therapeutic range (TTR). The secondary study outcomes were the mean INR variation over time, the number of warfarin dose changes needed, the difference between the predicted and the actual warfarin maintenance dose (the actual maintenance dose was defined as the stable warfarin dose associated with INR values within the therapeutic range on three consecutive measurements at least one week apart) evaluated along an extended follow-up of patients. Thromboembolic and bleeding complications were assessed during the first 30 days of treatment according to previous reports [1,21] and further detailed in S5 File. Since most extreme values of INR are associated with increased risk of thromboembolic and bleeding events, the incidence of INRs below 1.5 or above 4.0 were also recorded. Moreover, time to stable anticoagulation (defined as the first INR in a series of three INR within the therapeutic range) was also recorded over the 19 days observational period.

### Statistical analyses

Data on patients initiating warfarin in the local Thrombosis Centre [19,22] served to calculate the percentage of INR measures outside the therapeutic range during the study period in the control arm. This figure was set at 50%. To identify a difference of 10% between arms in the percentage of INR out of range with a 80% power and an α of 0.05 and assuming a standard deviation of 23% and a drop-out rate of 15%, 100 patients per group are needed to be enrolled.

Descriptive statistics are reported as appropriate; categorical data are expressed as frequencies (percentage) with their exact binomial 95% confidence intervals (CIs); continuous data are reported as mean and SD, or medians and ranges. Data were compared using the Fisher exact test or the Wilcoxon-rank sum test. The primary and secondary outcomes were analyzed adjusting for the possible confounding effects of age and BSA (Body Surface Area, DuBois & DuBois equation [Weight (kg) 0.425 x height (cm) 0.725 / 139.2]) categorized according to quartiles. Sensitivity analyses were performed to assess the primary outcomes differences between the two arms by alternative inclusion/exclusion of age and BSA in the statistical Poisson models. The number of out of range INRs was evaluated using a multivariate Poisson model. The TTR, calculated using the Rosendaal “step” method [23], was used as a dependent variable in the Poisson regression model. The same steps were taken to analyze the number of patients experiencing INR below 1.5 or above 4.0 and the time spent at INR below 1.5 or above 4.0.

The mean INR variation over time was assessed by Repeated Measured Analysis of Variance (RMANOVA). The number of dose changes were considered as count data and therefore evaluated by a multivariate Poisson model. Absolute error in warfarin weekly dose prediction was assessed by ANOVA. Statistical significance was set at p<0.05. All statistical analysis were performed using Stata version 12 (StataCorp, TX, USA) for Windows.

## Results

### Patients

**Fig 1:**
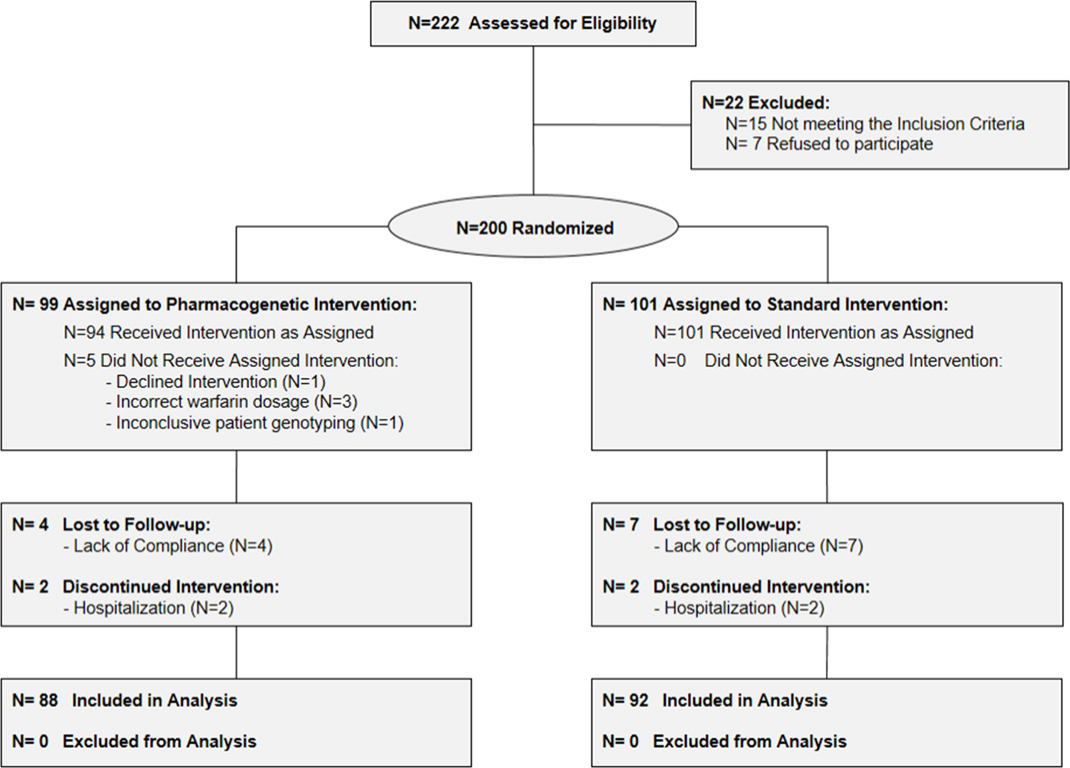
Study Flow diagram. –The diagram shows the progress through the phases of the randomized trial: enrolment, intervention allocation, follow-up, and data analysis.

Study ended on October 2012 when sample size goal was reached. Two hundred twenty-two consecutive patients were considered and two hundred patients were randomized (99 in the pharmacogenetic arm and 101 in the control arm). Eleven and nine patients were excluded from the study after randomization, thus the final cohort consisted of 88 and 92 in the pharmacogenetic and control arm, respectively (Fig 1). Patient baseline characteristics are outlined in Table 1. The differences in age and BSA in the two arms were taken into consideration in subsequent multivariate analyses. All other characteristics as well as other medications not known to interact with warfarin were well balanced between the two groups.

**Table 1:** Baseline patients’ characteristics in the two study arms.

Characteristic		PGX Arm	Control Arm
**Patients**, n		88	92
**Age**, yr, median (range)^a^		71 (39–84)	75 (48–88)
**Males,** n(%)		58 (65.9)	60 (65.2)
**BMI**, kg/m^2^, median (range)		26.89 (19.47–52.71)	29.79 (18.50–35.16)
**BSA**, m^2^,median (range)^b^		1.97 (1.51–2.45)	1.88 (1.50–2.29)
**Current smokers**, n (%)		8 (9.1)	10 (10.8)
**Current coffee drinkers**, n (%)		68 (77.2)	66 (71.7)
**Current alcohol drinkers**, n (%)		16 (18.2)	16 (17.4)
**CHADS2 score**, n (%)	0	16 (18.2)	12 (13.1)
	1	33 (37.5)	30 (32.6)
	2	25 (28.4)	37 (40.2)
	> 2	14 (15.9)	13 (14.1)
***CYP2C9***, n (%)	*1*1	52 (59.1)	59 (64.1)
	*1*2	17 (19.3)	20 (21.7)
	*1*3	12 (13.6)	8 (8.7)
	*2*2	2 (2.3)	2 (2.2)
	*2*3	2 (2.3)	2 (2.2)
	*3*3	3 (3.4)	1 (1.1)
***VKORC1–***1639, n (%)	GG	28 (31.8)	30 (32.6)
	GA	46 (52.3)	49 (53.3)
	AA	14 (15.9)	13 (14.1)
***CYP4F2***, n (%)	*1*1	38 (43.2)	43 (46.7)
	*1*3	38 (43.2)	36 (39.2)
	*3*3	12 (13.6)	13 (14.1)
**Follow-up,** d, median (range)		397 (30–1037)	359 (30–984)

^a^p = 0.023 for age

^b^p = 0.009 for BSA. PGX = pharmacogenetic; BMI = Body Mass Index calculated using the Quetelet formula [Weight (kg) / height (m)^2^]; BSA = Body Surface Area calculated using the DuBois & DuBois equation [Weight (kg) 0.425 x height (cm) 0.725 / 139.2]; Patients were classified as smokers or non-smokers and coffee or non-coffee-drinkers based on their current habits, irrespective of the number of cigarettes smoked or cups of coffee drunk daily. Patients who currently consumed 20grams of alcohol or more per day were considered alcohol consumers. CHADS2 score was calculated as described elsewhere [24] taking into account Congestive heart failure, Hypertension, Age ≥75 years, Diabetes mellitus and prior Stroke or transient ischemic attack; *CYP2C9* and *CYP4F2* alleles were defined according to “The Human Cytochrome P450 (CYP) Allele Nomenclature Database” at http://www.cypalleles.ki.se/.

### Primary outcomes

The primary outcome analysis involved all patients of the final cohort.

No statistically significant difference was found in the number of INRs outside the therapeutic range in the two study arms (p = 0.79), being total INRs out of range 45.1% (95% CI 40.4–49.7) and 43.6% (95% CI 38.7–48.6) in the pharmacogenetic and control arm respectively. The number of INRs outside the therapeutic range were not associated with age and BSA (p = 0.76 and p = 0.75 respectively).

**Fig 2:**
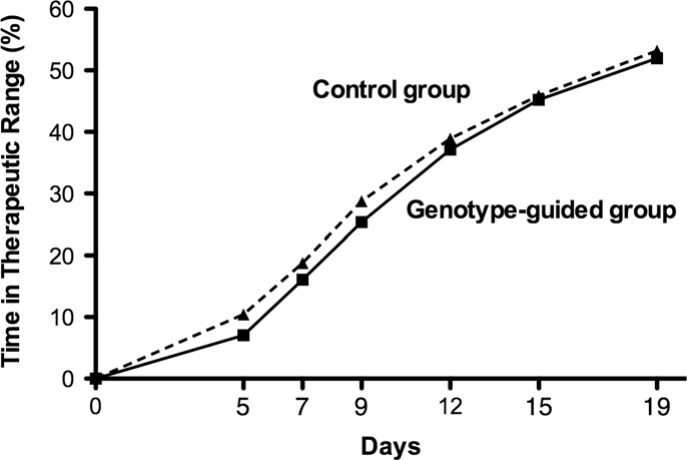
Percentage of Time in the Therapeutic INR Range according to subgroup. The percentage of time in the therapeutic INR range for pharmacogenetic arm (Genotype guided group) and control arm (Control group) are shown over the 19 day observational period.

Fig 2 shows the mean TTR, over the 19 days observational period, for pharmacogenetic and control arm. At day 19 of treatment mean TTR was 51.9% (95% CI 48.4–55.5) in the pharmacogenetic and 53.2% (95% CI 48.9–57.4) in the control arm and was not statistically different (p = 0.71).

Mean TTR was not associated with age and BSA (p = 0.70 and p = 0.66 respectively).

Sensitivity analyses for both primary outcomes showed no statistically significant differences by alternative inclusion/exclusion of age and BSA in the statistical Poisson models.

At ancillary analysis the distribution of INRs below 1.5, over the first 19 days of treatment, was not significantly different between arms (Bonferroni adjusted p = 0.12) although the number of patients experiencing at least one INRs below 1.5 was lower in the pharmacogenetic (27/88) (30.7%; 95% CI 21.3–41.4) with respect to the control arm (46/92) (50.0%; 95% CI 39.4–60.6). The distribution of INRs above 4.0 were not significantly different in the two arms (Bonferroni adjusted p = 0.32) although the number of patients experiencing at least one INRs above 4.0 tended to be lower in the pharmacogenetic (4/88) (4.5%; 95% CI 1.3–11.2) than in the control arm (8/92) (8.7%; 95% CI 3.8–16.4). Only two severe over-anticoagulation episodes (INR>6.0) were recorded over the 19 days observational period of the study, they were both experienced by a single patient assigned to the control arm whose actual maintenance dose was 6.25 mg/week. Considering only patients potentially at higher risk of over-anticoagulation i.e those requiring low warfarin maintenance doses (lower than 26.25 mg/week), the distribution of INRs above 4.0, over the first 19 days of treatment, was not significantly different between arms (Bonferroni adjusted p = 0.16) although the number of patients with at least one INR>4.0 was lower in the pharmacogenetic (2/26) (7.7%; 95% CI 1.0–25.1) than in the control arm (7/28) (25.0%; 95% CI 10.7–44.9).

Overall, the percentage of time spent at INR>4.0 was significantly lower in the pharmacogenetic (0.7%, 95% CI 0.4–1.4) than in the control arm (1.8%, 95% CI 0.4–3.3) (Bonferroni adjusted p = 0.02). The percentage of time spent at INR<1.5 was not significantly different (Bonferroni adjusted p = 0.96).

### Secondary outcomes

The secondary outcome analysis involved all patients of the final cohort unless otherwise specified.

**Fig 3:**
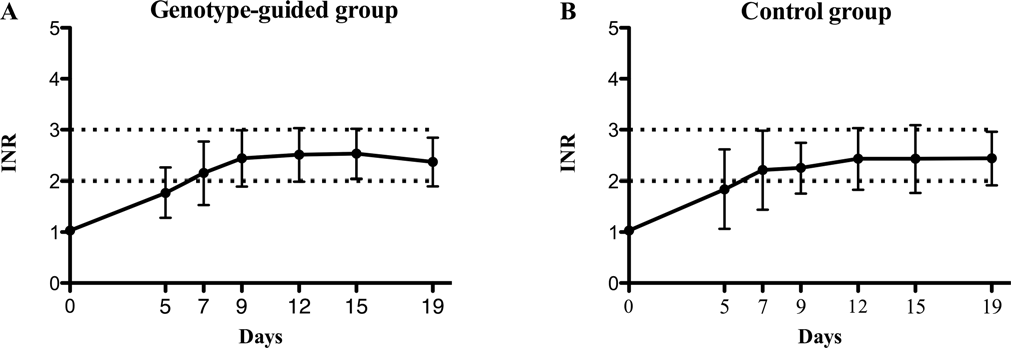
Overall mean INR variations according to subgroup. Comparison of finding in the pharmacogenetic arm (section A, Genotype guided group) and control arm (section B, Control group) during the 0–19 day time frame. The bars denote Standard Deviations, the dotted lines indicate the therapeutic margins. INR = International Normalized Ratio.

Fig 3 shows variations over time of mean INR values in the two treatment arms. Data, adjusted for age and BSA, yielded no significant differences (p = 0.75).

Number of dose adjustments was not different between the arms (p = 0.77).

A total of 120 patients reached stable anticoagulation within the 19 days time, 60 belonged to pharmacogenetic and 60 to control arm (χ^2^ = 0.177; p = 0.67).

Stable anticoagulation was not significantly different in the pharmacogenetic and control arms both considering the mean time required to reach it (5.96 days (95% CI 5.00–9.93 days) and 5.05 (95% CI 4.24–5.86) respectively) (p = 0.28) and the mean therapeutic dosage needed when stable anticoagulation was achieved (4,40 mg/die (95% CI 3.93–4.89 mg/die) and 4.13 mg/die (95% CI 3.75–4.51) respectively) (p = 0.86).

In 155/180 patients, 75 randomized to the pharmacogenetic arm and 80 to the control arm (χ^2^ = 0.112, p = 0.737) actual weekly maintenance dose could be determined. In this subset of patients the mean absolute error in warfarin weekly dose prediction was not significantly different in the pharmacogenetic and control arms: 11.28 mg/week (95% CI 8.79–13.77 mg/week) and 9.85 mg/week (95% CI 7.59–12.11 mg/week) respectively with a difference of 1.43 mg/week (95% CI -1.91 to 4.76 mg/week) (p = 0.95). No major/minor thromboembolic or bleeding complications were recorded during the 0–30 daytime period.

## Discussion

A validated pharmacodynamic-based approach has been used since several years in our Institution to predict warfarin maintenance doses in patients initiating anticoagulant treatment [19]. Recent observational studies suggest that anticoagulation control may be successfully improved by genotype-guided warfarin dosing [2,7]. We have previously demonstrated that a pharmacogenetic algorithm, based on *VKORC1*, *CYP2C9* and *CYP4F2* gene variants, was more accurate than others among Italian Caucasian patients [18]. This result prompted us to design and conduct this randomized prospective study aimed to test the benefit of genotype- versus pharmacodynamic-based warfarin dosing. The choice of primary outcomes was based on the knowledge that low or high INR values during warfarin initiation period are the major factors associated to inappropriate dose changes exposing patients at a higher risk of adverse events [1,25–27].

The percentage of total INRs out of range in the pharmacogenetic arm (45%) was close to that in the control arm (44%). Accordingly, mean TTR in pharmacogenetic-guided warfarin dosing (52%) was similar to that in the control arm (53%). These results do not support the superiority of genotype-guided warfarin dosing. Possible explanations are related to the fact that our pharmacodynamic-based nomogram model is highly accurate and that the advantage of the pharmacogenetic approach may be limited to the first 6 days as dose adaptations were carried out following the same standard procedures in both study arms from day 7 onwards. On the other hand, the pharmacogenetic algorithm accounts for ~50% of warfarin dose variability meaning that other relevant environmental factors interfere with the control of anticoagulation.

These considerations might also explain the different results in our trial with respect to EU-PACT and COAG studies. At day 19 of treatment, mean TTR in the present and the EU-PACT (constructed out from EUPACT Fig 1B) trials were similar in the pharmacogenetic arms (52% and 50% respectively), but quite different in the control arms (53% and 40%, respectively). Since timing of patients’ INR measurements and clinical visit were comparable in both trials, it is reasonable to hypothesize that the pharmacodynamic warfarin dosing scheme in the present study was more accurate than the standard warfarin dosing in the EU-PACT control arm. This might be related to the higher loading-dose regimen (10mg/die vs 5mg/die) leading to excessive anticoagulation in EU-PACT trial [9].

The TTR mean difference between arms (genotype-guided arm minus control arm) after 19 days was confirmed extending the observational period to 28 days being mean TTR in the present trial 60% both in genotype-guided and control arm while in the EU-PACT study they were 55% and 46% respectively. This is not unexpected and in agreement with findings of trials evaluating genotype-guided warfarin initiation dosing [15,16]. Our algorithm was designed to start warfarin dosing and therefore it is likely to display its effects in the very early phases of treatment.

A direct comparison of TTR at day 28 was also possible for COAG trial and mean TTR was higher in our than in COAG study both for genotype-guided (60% vs 45%) and control group (60% vs 45%). This finding may arise from differences in the quality of genotype-guided algorithm and in the prescription skills of trained physician of a single centre thereafter. It may also depend on indication for anticoagulation treatment, study design and population ethnic background [28].

Upon considering the control of over- and under-anticoagulation, no significant difference was evidenced in the rate of patients with INR < 1.5, but the time they spent at INR>4.0 was significantly lower in the pharmacogenetic arm suggesting a better accuracy of a pharmacogenetic algorithm for estimating the appropriate initial and maintenance dose particularly in patients requiring very low warfarin dosages.

The reported results suggest that genotype-guided warfarin dosing, when compared to accurate clinical standard of care, has a marginal clinical utility. The possible clinical utility of pharmacogenetic based dosing is worth being evaluated in less carefully managed standard of cares settings [28].

The secondary endpoints of our study showed a comparability of the anticoagulation schemes adopted. The overall INR variations over time, the number of warfarin dose changes, the time to stable anticoagulation and the difference between the predicted and the actual warfarin maintenance dose were in fact not significantly different in the two arms of the trial. Importantly, no thromboembolic or major/minor bleeding events were recorded in either arm; however, the present trial was not adequately powered to detect possible differences for this outcome, a limitation shared with previous trials. With respect to COAG and EU-PACT trials we randomized a lower number of patients and this might be a limitation. However our study is strengthened by the fact that: 1) the first dose of warfarin was informed by genotyping in all patients allocated to the pharmacogenetic arm and 2) genotype-guided dosing was compared with the local standard of care. Generalisabilty to subjects of Caucasian ethnicity is possible since the studied patients were Italian Caucasian.

## Conclusions

This study demonstrates that a pharmacogenetic algorithm is not superior to an accurate clinically-adopted pharmacodynamic based nomogram in terms of the number of INRs outside the therapeutic range. The former allow a slightly better control of over-anticoagulation, which is particularly relevant in specific subsets of patients such as those requiring very low warfarin doses. It remains to be defined whether the benefits of the translation of these findings into clinical practice are cost-effective.

## Supporting Information

S1 CONSORT Checklist (DOCX)

S1 File Inclusion and exclusion criteria. (DOCX)

S2 File Genotyping procedure. (DOCX)

S3 File Pharmacodynamic nomogram. (DOCX)

S4 File Pharmacogenetic dosing algorithms. (DOCX)

S5 File Definition of adverse events. (DOCX)

S6 File Trial Protocol. (DOCX)
