# Supplementary material for: A Randomized Trial of Pharmacogenetic Warfarin Dosing in Naïve Patients with Non-Valvular Atrial Fibrillation
Source: PLoS One. 2015 Dec 28;10(12):e0145318. doi: 10.1371/journal.pone.0145318 (PMC4692529; doi:10.1371/journal.pone.0145318)
Supplement: S1 File — (DOCX) [file pone.0145318.s002.docx]

**INCLUSION AND EXCLUSION CRITERIA**

Inclusion criteria:

- Age >18 years
- Atrial fibrillation
- Warfarin-naïve patients with indication for warfarin treatment with target INR between 2.0 and 3.0

Exclusion Criteria:

- Pregnancy (present or planned)
- Drug interactions (amiodarone, rifampin and carbamazepine)
- Basal INR > 1.2
- Unwillingness to sign an informed consent statement.
