# Supplementary material for: A Randomized Trial of Pharmacogenetic Warfarin Dosing in Naïve Patients with Non-Valvular Atrial Fibrillation
Source: PLoS One. 2015 Dec 28;10(12):e0145318. doi: 10.1371/journal.pone.0145318 (PMC4692529; doi:10.1371/journal.pone.0145318)
Supplement: S2 File — (DOCX) [file pone.0145318.s003.docx]

**GENOTYPING PROCEDURES**

Genomic DNA was extracted from 200 μL whole blood samples by means of the BioSprint 15 DNA Blood Kit (Qiagen, Milan, Italy) using semi-automated magnetic particle technology (KingFisher mL; ThermoLabsystems, Vantaa, Finland). Genetic analyses of rs9923231 (*VKORC1* -1639G>A), rs1799853 and rs1057910 (*CYP2C9 *1*, **2* and **3* alleles) and of rs2108622 (*CYP4F2 *1* and **3* alleles) were performed on the Real Time PCR instrumentation ABI Prism 7900 HT by means of Taqman Drug Metabolisms Assays (C_30403261_20, C_25625805_10, C_27104892_10 and C_16179493_40 respectively) following the manufacturer’s instructions (Life technologies, Italy). *CYP2C9* and *CYP4F2* allele assignment was performed according to the Human Cytochrome P450 (CYP) Allele Nomenclature Committee (<http://www.imm.ki.se/CYPalleles/>). After the completion of genetic analyses DNA samples were stored at -20°C.
