# Supplementary material for: A Randomized Trial of Pharmacogenetic Warfarin Dosing in Naïve Patients with Non-Valvular Atrial Fibrillation
Source: PLoS One. 2015 Dec 28;10(12):e0145318. doi: 10.1371/journal.pone.0145318 (PMC4692529; doi:10.1371/journal.pone.0145318)
Supplement: S3 File — (DOCX) [file pone.0145318.s004.docx]

**Pharmacodynamic nomogram**

The predicted weekly maintenance dose on the Basis of INR on Day 5 After 5 mg/day of warfarin for four consecutive days are detailed in the following table^a^

| **INR on Day5** | **mg/wk** |
| --- | --- |
| 1.0 | 71 |
| 1.1 | 57 |
| 1.2 | 48 |
| 1.3 | 43 |
| 1.4 | 39 |
| 1.5 | 35 |
| 1.6 | 33 |
| 1.7 | 31 |
| 1.8 | 29 |
| 1.9 | 27 |
| 2.0 | 26 |
| 2.1 | 24 |
| 2.2 | 23 |
| 2.3 | 22 |
| 2.4 | 21 |
| 2.5 | 20 |
| 2.6 | 19 |
| 2.7 | 18 |
| 2.8 | 17 |
| 2.9 | 16.5 |
| 3.0 | 16 |
| 3.1 | 15 |
| 3.2 | 14 |
| 3.3 | 13.5 |
| 3.4 | 13 |
| 3.5 | 12 |
| 3.6 | 11.5 |
| 3.7 | 11 |
| 3.8 | 10.5 |
| 3.9 | 10 |
| 4.0 | 9 |
| 4.1 | 8.5 |
| 4.2 | 8 |
| 4.3 | 7.5 |
| 4.4 | 7 |

^a^According to this scheme, a warfarin maintenance dose of 20 mg/week is predicted for a patient with an INR of 2.5 on day 5.
