# Supplementary material for: A Randomized Trial of Pharmacogenetic Warfarin Dosing in Naïve Patients with Non-Valvular Atrial Fibrillation
Source: PLoS One. 2015 Dec 28;10(12):e0145318. doi: 10.1371/journal.pone.0145318 (PMC4692529; doi:10.1371/journal.pone.0145318)
Supplement: S4 File — (DOCX) [file pone.0145318.s005.docx]

**PHARMACOGENETIC DOSING ALGORITHMS**

Loading Dose Algorithm

The personalized loading dose (LD) was calculated using the standard formula for the one-compartment pharmacokinetic model:

*LD (mg) = volume of distribution (l) × target steady state plasma concentration (mg/l).*

The volume of distribution was estimated on the basis of body weight (0.14 l/kg) [1].

The ideal steady state plasma concentration (Css) target, which should theoretically produce an INR in the middle of the therapeutic range (namely 2.5), was calculated on the basis of the *VKORC1* genotype according to a PK-PD model described elsewhere [2], as follows:

a) 0.25 mg/ml for *VKORC1 AA*;

b) 0.34 mg/ml for *VKORC1 GA*;

c) 0.52 mg/ml for *VKORC1 GG*.

Maintenance Dose Algorithm

The estimated weekly maintenance dose (MD) was:

*MD (mg/week) = [7.39764 – (0.02734 x age) + (1.06287 x BSA) – (1.04468 for VKORC1 -1639 AG) – (2.12117 for VKORC1 -1639 AA) – (0.78983 for CYP2C9*1*2) – (1.17138 for CYP2C9 *1*3) – (1.81292 for CYP2C9 *2*2 or *2*3 or *3*3) – (0.46723 for CYP4F2 *1*3) – (0.71528 for CYP4F2 *1*1)]^2^.*

where:

age is entered as years, BSA (Body Surface Area) is calculated as [Weight(kg)0.425 x height(cm)0.725/139.2]. *VKORC1* -1639 AG, *VKORC1* -1639 AA, *CYP2C9*1*2, CYP2C9 *1*3, CYP2C9 *2*2* or **2*3* or **3*3, CYP4F2 *1*3* and *CYP4F2 *1*1* are coded 0 if absent and 1 if present.

**References**

1. Goodman & Gilman’s. The Pharmacological Basis of Therapeutics. 12^th^ ed. New York: McGraw Hill; 2011.
2. Hamberg AK, Dahl ML, Barban M, Scordo MG, Wadelius M, Pengo V, et al. A PK-PD model for predicting the impact of age, CYP2C9, and VKORC1 genotype on individualization of warfarin therapy. Clin Pharmacol Ther. 2007; 81: 529-538.
