# Supplementary material for: A Randomized Trial of Pharmacogenetic Warfarin Dosing in Naïve Patients with Non-Valvular Atrial Fibrillation
Source: PLoS One. 2015 Dec 28;10(12):e0145318. doi: 10.1371/journal.pone.0145318 (PMC4692529; doi:10.1371/journal.pone.0145318)
Supplement: S5 File — (DOCX) [file pone.0145318.s006.docx]

**DEFINITIONS OF ADVERSE EVENTS**

Bleeding complications were classified according to the Italian Study on Complications of Oral Anticoagulant Therapy (ISCOAT) [1]:

“Major bleeding: fatal (death due to haemorrhage); intracranial (documented by imaging), ocular (with blindness), articular, or retroperitoneal; if surgery or angiographic intervention was required to stop bleeding; and if bleeding led to haemoglobin reduction of 2 g/dl or more and/or need for transfusion of two or more blood units.

Minor bleeding was all cases of bleeding not classified as major.

Non-relevant bleeding was bruising, small ecchymoses or epistaxis, occasional haemorrhagic bleeding, or microscopic haematuria” [1].

Thromboembolic complications were defined according to the Italian Study on Complications of Oral Anticoagulant Therapy (ISCOAT) [2]:

Major thrombotic events were considered deep vein thrombosis (first event or recurrence, in whatever site), pulmonary embolism, acute myocardial infarction, stroke, peripheral or visceral arterial thromboembolism, clinical of peripheral obliterative arterial disease leading to amputation or reconstructive surgery.

Minor thrombotic events were considered cerebral transient ischemic attacks and superficial thrombophlebitis.

**References**

1. Palareti G, Leali N, Cocchieri S, Poggi M, Manotti C, D'Angelo A, et al. Bleeding complications of oral anticoagulant treatment: an inception-cohort, prospective collaborative study (ISCOAT). Italian Study on Complications of Oral Anticoagulant Therapy. Lancet. 1996; 348: 423-428.
2. Palareti G, Manotti C, D’Angelo A, Pengo V, Erba N, Moia M, et al. Thrombotic events during oral anticoagulant treatment: results of the inception-cohort, prospective, collaborative ISCOAT study: ISCOAT study group (Italian Study on Complications of Oral Anticoagulant Therapy). Thromb Haemost*.* 1997; 78: 1438- 1443.
