# Supplementary material for: A Randomized Trial of Pharmacogenetic Warfarin Dosing in Naïve Patients with Non-Valvular Atrial Fibrillation
Source: PLoS One. 2015 Dec 28;10(12):e0145318. doi: 10.1371/journal.pone.0145318 (PMC4692529; doi:10.1371/journal.pone.0145318)
Supplement: S6 File — (DOCX) [file pone.0145318.s007.docx]

**trial PROTOCOL**

**Title**

Early identification of warfarin maintenance dose

Warfarin, the most prescribed oral anticoagulant, in Italy, in the year 2008 approximately 6,500,000 subjects were estimated to be on warfarin, the annual increase being 5%. Warfarin has a very narrow therapeutic window and wide and unpredictable inter-individual variability in dose-response leading to a difficult achievement and control of its antithrombotic effect.

This orally administered drug is a racemic mixture of S- and R-warfarin that differ in terms of their pharmacokinetic and pharmacodynamic properties. S-warfarin is by far more pharmacologically active than R-warfarin, is largely absorbed (>90%) after oral administration and is inactivated in the liver by cytochrome CYP2C9. The activity of this enzyme is extremely variable in the population mainly due to the presence of 2 allelic variants (CYP2C9*2 and CYP2C9*3) associated with reduced activity compared to the common wild-type allele (CYP2C9*1). The combination of these 3 main alleles gives rise to 6 different genotypes associated with a decreasing enzymatic activity: *1*1>*1*2>*1*3>*2*2>*2*3>*3*3 [1]. Moreover many pharmacological interactions have been reported resulting either in the induction or inhibition of cytochrome P450 2C9 itself.

Similarly complex is the relation between concentration of the drug and its effect since the gene encoding for the target of warfarin – Vitamin K epoxide reductase- is also polymorphic and certain genetic variants (VKORC1 1173 C>T and -1639G>A in complete linkage [2]) have been demonstrated to been associated with reduced warfarin requirements [3]. Since warfarin competes with vitamin K for the binding to vitamin K epoxide reductase, the food intake of vitamin K is an important factor modulating the anticoagulant effect.

A further aspect of these complex phenomena is the lag time between drug administration and the onset of the antithrombotic activity which depends on the progressive decrease of the vitamin K dependent coagulation factors (II, VII, IX and X).

Moreover a recent study demonstrated that a genetic polymorphism (rs2108622; V433M) in cytochrome P450 4F2 (CYP4F2) was associated with warfarin dose [4] .

Currently warfarin maintenance dose is individually adjusted after a standard initial dosing on the basis of the international normalized ratio (INR) monitoring protocols (trial and error schemes). During the initial phase of the treatment, often lasting weeks or even months, patients may be at risk of thrombotic (drug under-dosing) and bleeding (drug over-dosing) events. The scheme for warfarin treatment validated [5] and currently adopted by our Institution is based on the consecutive administration of a 5 mg warfarin dose the first 4 days and the INR measurement at day 5. On the basis of the hyperbolic correlation existing between day 5 INR and warfarin maintenance dose it is possible to calculate the weekly warfarin dose of the drug (Table 1).

The attending physician prescribes subsequent adjustment in warfarin dosing with the assistance of the PARMA v5.7 software [6].

During the last years several research groups [4, 7-10] have tried to determine which demographic (age, sex, weight, height, etc.) and genetic factors (VKORC1, CYP2C9 and CYP4F2)associated with warfarin maintenance dose might be useful in predicting initial drug dosing. These studies demonstrated that the pharmacogenetic approach can predict 50% of the inter-individual variability if warfarin dosing.Our Group has recently developed, in a retrospective study, a pharmacogenetic algorithm, based on patients’ age and body surface area (BSA) and *VKORC1*, *CYP2C9*, *CYP4F2* polymorphisms. This algorithm could explain 55% of warfarin dose variability and allowed the correct prediction of warfarin dose regimens in the great majority of the Italian patients. VKORC1 genotype information can also be used to predict the personalized loading dose of warfarin in order to achieve more rapidly the optimal anticoagulation (INR 2.5).

The study is aimed to verify if patients with atrial fibrillation may benefit in term of anticoagulation control and safety from the advanced prediction of their personalized warfarin loading and maintenance dosage with respect to patients treated according to the standard care (a posteriori adjustment) adopted in our Institution.

**STUDY DESIGN:**

The study will enroll warfarin-naïve patients with atrial fibrillation and indication for warfarin treatment with target INR between 2.0 and 3.0.

Patients will be randomized to receive initial warfarin doses either established according to the standard care (control group) or determined by the pharmacogenetic algorithm (intervention group) according to individual age, BSA and *CYP2C9, CYP4F2*and*VKORC1* genotypes.

Patients will be selected based on the following eligibility criteria:

Inclusion criteria:

- Age >18 years
- Non-valvular atrial fibrillation
- Warfarin-naïve patients with indication for warfarin treatment with target INR between 2.0 and 3.0

Exclusion Criteria:

- Pregnancy (present or planned)
- Treatment with drugs interfering with warfarin (Amiodarone, rifampin and carbamazepine)
- Basal INR > 1.2
- Unwillingness to sign an informed consent statement.

Primary outcome measuresof the study are:

- percentage of INR out of range (INR<2 or>3) of each patient[ Time Frame: Day 0-19 ];
- time in therapeutic range (TTR) defined as the fraction of the study time (days) spent within the therapeutic range (INR 2-3) by patients belonging to the two arms [11][Time Frame: Day 0-19 ];

Secondary outcome measures of the study are:

- number of changes in warfarin dosage [ Time Frame: Day 0-19 ] ;
- difference between predicted and actual warfarin maintenance dose [ Time Frame: Day 19 ];
- thromboembolic and Bleeding complications [ Time Frame: Day 0-30 ].

**STUDY PROCEDURES**

Patients enrollment will be performed from Monday to Wednesday at the Anticoagulation Clinic of the University Hospital of Padova. Fully informed consent will be obtained in writing from all the participants.

The day of enrolment clinical data will be recorded and 15mL of peripheral blood will be collected (for routine testing and genetic analyses). Patients accepting to participate in the study will be randomly assigned to control group or intervention group. The assignment to the study groups will be performed by block randomization . On Wednesday genetic analyses of rs9923231 (*VKORC1* -1639G>A), rs1799853 and rs1057910 (*CYP2C9 *1*, **2* and **3* alleles) and of rs2108622 (*CYP4F2 *1* and **3* alleles) will be performed for each patients at the Department of Laboratory Medicine of the University Hospital of Padova by means of Real Time PCR. Results will be available within Thursday morning in order to calculate the predicted warfarin maintenance dose of patients in the intervention group by means of the pharmacogenetic algorithm. From the day of enrolment till the initiation of treatment patients will be prescribed Enoxaparin (4000 IU q.d. subcutaneously).On Thursday patients of both arms will start treatment (day 1). During the observational period of the study, which encompasses the first 19 days of the treatment, patients will be examined at the Anticoagulation Clinic of the University Hospital of Padova at day 5, 7, 9, 12, 15, 19. At each time point INR will be measured to monitor the intensity and control of anticoagulation. The study flow chart is reported in figure 1.

Control Group (control arm)

Patients treated according to standard care will be administered 5mg warfarin at 8 p.m. for 4 consecutive days (day 1 to day 4 ) and INR value detected at day 5 (Monday, INR1) will be used to establish the warfarin dosing according to table 1. Five following visit will be scheduled for each patient at day 7 (Wednesday, INR 3), 9 (Friday, INR 5) of the first week of treatment, at day 12 (Monday INR 8), 15 (Thursday, INR 11) of the second week of treatment and finally at day 19 (Monday, INR 15) of the third week of treatment. Each patients will receive a timetable of the scheduled follow-up. The attending physician will be allowed to perform further visits on the basis of safety considerations. At each visit a peripheral blood sample will be collected from patients for INR measurement.

Intervention group (PGX Arm)

Patients treated according to the pharmacogenetic scheme will be administered, at 8 p.m., in the first four days of treatment (day 1 to day 4) warfarin dosage will be calculated on the basis of pharmacogenetic results. The first day each patients will be administered a loading dose calculated on the basis of his weight and VKORC1 genotype according to the following scheme:

| genotype | Loading dose (mg) |
| --- | --- |
| VKORC1 AA | 0.25 ×weight× 0.14 |
| VKORC1 GA | 0.34 ×weight× 0.14 |
| VKORC1 GG | 0.52 ×weight× 0.14 |

This table is derived from the formula for the one-compartment pharmacokinetic model:

Loading Dose (LD) (mg) = volume of distribution (L) × target steady state plasma concentration (mg/L).

The volume of distribution will be estimated on the basis of body weight (0.14 L/Kg) [12], while the target steady state plasma concentration (mg/L) will be calculated according to a pharmacokinetic-pharmacodynamic model described by Hamberg and colleagues [13]

a) 0.25 mg/mL for *VKORC1 AA*;

b) 0.34 mg/mL for *VKORC1 GA*;

c) 0.52 mg/mL for *VKORC1 GG*.

In any case the maximum loading dose to be administered will not be higher than 10mg for safety reason.

From day 2 till day 6 of treatment warfarin dosages will be calculated on the basis of age , Body Surface Area (BSA) and CYP2C9, CYP4F2 and VKORC1 according to the following pharmacogenetic algorithm:

Dose di mantenimento = [7.40 – 0.027(age) + 1.06(BSA) – 1.04(VKORC1 AG) – 2.12(VKORC1)AA) – 0.79(CYP2C9 *1*2) – 1.17(CYP2C9 *1*3)

– 1.81(CYP2C9 *2*2+*2*3+*3*3) – 0.71(CYP4F2 CC) – 0.47(CYP4F2 CT)]**^0.5^**

From day 7 of treatment (Wednesday, INR 3) onward the attending physician on the basis of INR results and his clinical experience will adjust warfarin dosage according to standard care as in the control group of patients. Each patients will receive a timetable of the scheduled follow-up. The attending physician will be allowed to performed further visits on the basis of safety considerations. At each visit a peripheral blood sample will be collected from patients for INR measurement.

Statistical analysis

Statistical analysis will be specified and planned prior to any comparative analysis. We exclude an on-going interim analysis.

For both primary and secondary endpoints, it is considered appropriate to test the null hypothesis by a two side α levels.

Before proceeding with inferential analyses, the data will be fully inspected by an exploratory analysis. Descriptive statistics will be used to inspect data; categorical data will be expressed as frequencies and the 95% confidence intervals will be calculated by the exact binomial methods. For continuous data, normality will be at priori assessed by Shapiro Wilk test and by using Q-Q plots. Therefore, mean and standard deviation or medians and ranges will be alternatively used to summary results of continuous data. Differences in potentially clinical covariates (e.g., Age, gender, BMI, BSA, etc.) will be assessed by Student’s t-test (for continuous data) or by Fisher exact test (for frequencies comparison).

For the primary and secondary endpoints, the differences between arms will be assessed by appropriate statistics, according to data distribution.

Sample size calculation

Bases on data from the Thrombosis Center local database, we expect that, during the study period, patients in control arm will experience a percentage of INR measures outside the therapeutic range will be 50%. In order to demonstrate a 10% of improvement in the percentage of INR out of range for the Pharmacogenetic over the Control arm, with a 80% of power, at a 5% significance level, a 23% standard deviation and a drop-out rate of 15%, 100 patients per group will be needed for each arm.

**REFERENCES**

1. Scordo MG, Pengo V, Spina E, Dahl ML, Gusella M, Padrini R. Influence of CYP2C9 and CYP2C19 genetic polymorphisms on warfarin maintenance dose and metabolic clearance. Clin Pharmacol Ther 2002; 72: 702-710.
2. Crawford DC, et al. Identifying the genotype behind the phenotype: a role model found in VKORC1 and its association with warfarin dosing. Pharmacogenomics 2007; 8:487-496.
3. D’Andrea G, D’Ambrosion RL, Di Perna P, Chetta M, Santacroce R, Brancaccio V, Grandone E, Margaglione M. A polymorphism in the VKORC1 gene is associated with an interindividual variabilità in the dose-anticoagulant effect of warfarin. Blood 2005; 105: 645-649.
4. Caldwell MD, Awad T, Johnson JA, Gage BF, Falkowski M, Gardina P, Hubbard J, Turpaz Y, Langaee TY, Eby C, King CR, Brower A, Schmelzer JR, Glurich I, Vidaillet HJ, Yale SH, Qi Zhang K, Berg RL, Burmester JK. CYP4F2 genetic variant alters required warfarin dose. Blood. 2008 15;111(8):4106-12.
5. Pengo V, Biasiolo A, Pegoraro C. A simple scheme to initiate oral anticoagulant treatment in outpatients with nonrheumatic atrial fibrillation. Am J Cardiol 2001; 88: 1214-1216.
6. Manotti C, Moia M, Palareti G, et al. Effect of computer-aided management on the quality of treatment in anticoagulated patients: a prospective, randomized,multicenter trial of APROAT (Automated PRogram for Oral Anticoagulant Treatment). Haematologica 2001;86:1060-70.
7. Sconce EA, Khan TI, Wynne HA, Avery P, Monkhouse L, King BP, Wood P, Kesteven P, Daly AK, Kamali F. The impact of CYP2C9 and VKORC1 genetic polymorphism and patient characteristics upon warfarin dose requirements: proposal for a new dosing regimen. Blood 2005; 106: 2329-2333.
8. Takahashi H, Wilkinson GR, Nutescu EA, Morita T, Ritchie MD, Scordo MG, Pengo V, Barban M, Padrini R, Ieiri I, Otsubo K, Kashima T, Kimura S, Kijima S, Echizen H. Different contributions of polymorphisms in VKORC1 and CYP2C9 to intra- and inter-population differences in maintenance dose of warfarin in Japanese, Caucasians and African-Americans. Pharmacogenet Genomics. 2006;16:101-10.
9. Hamberg AK, Dahl ML, Barban M, Scordo MG, Wadelius M, Pengo V, Padrini R, Jonsson EN. A PK-PD model for predicting the impact of age, CYP2C9, and VKORC1 genotype on individualization of warfarin therapy. Clin Pharmacol Ther. 2007 ;81:529-38.
10. Kimura R, Miyashita K, Kokubo Y, Akaiwa Y, Otsubo R, Nagatsuka K, Otsuki T, Okayama A, Minematsu K, Naritomi H, Honda S, Tomoike H, Miyata T. Genotypes of vitamin K epoxide reductase, γ-glutamyl carboxylase, and cytochrome P450 2C9 as determinants of daily warfarin dose in Japanese patients. Thromb Res 2007; 120: 181-186.
11. Rosendaal FR, Cannegieter SC, van der Meer FJ, Briët E. A method to determine the optimal intensity of oral anticoagulant therapy. ThrombHaemost 1993;69:236-9.
12. Goodman &Gilman’s The Pharmacological Basis of Therapeutics, 12^th^ edition. Brunton LL, Chabner BA, Knollmann BC Editors, McGraw Hill New York, 2011.
13. Hamberg AK, Dahl ML, Barban M, et al. A PK-PD model for predicting the impact of age, CYP2C9, and VKORC1 genotype on individualization of warfarin therapy. Clin Pharmacol Ther. 2007;81:529-38.

TABLE 1. Standard warfarin dosing after day 5 INR measurement.

| INR | mg/week |  | Quarters (1.25 mg) of pill (5 mg) | | | | | | |  |  |
| --- | --- | --- | --- | --- | --- | --- | --- | --- | --- | --- | --- |
| day 5 |  |  | lun | mar | mer | gio | ven | sab | dom |  | mg/week |
|  |  |  |  |  |  |  |  |  |  |  |  |
| 1 | 71 |  | 9 | 8 | 8 | 8 | 8 | 8 | 8 |  | 71.25 |
| 1.1 | 57 |  | 7 | 6 | 7 | 6 | 7 | 6 | 7 |  | 57.5 |
| 1.2 | 48 |  | 6 | 5 | 6 | 5 | 5 | 5 | 6 |  | 47.5 |
| 1.3 | 43 |  | 5 | 5 | 5 | 5 | 5 | 4 | 5 |  | 42.5 |
| 1.4 | 39 |  | 5 | 4 | 4 | 5 | 4 | 4 | 5 |  | 38.75 |
| 1.5 | 35 |  | 4 | 4 | 4 | 4 | 4 | 4 | 4 |  | 35 |
| 1.6 | 33 |  | 4 | 3 | 4 | 4 | 4 | 3 | 4 |  | 32.5 |
| 1.7 | 31 |  | 4 | 3 | 4 | 3 | 4 | 3 | 4 |  | 31.25 |
| 1.8 | 29 |  | 4 | 3 | 3 | 4 | 3 | 3 | 3 |  | 28.75 |
| 1.9 | 27 |  | 3 | 3 | 3 | 4 | 3 | 3 | 3 |  | 27.5 |
| 2 | 26 |  | 3 | 3 | 3 | 3 | 3 | 3 | 3 |  | 26.25 |
| 2.1 | 24 |  | 3 | 2 | 3 | 3 | 3 | 2 | 3 |  | 23.75 |
| 2.2 | 23 |  | 3 | 2 | 3 | 2 | 3 | 2 | 3 |  | 22.5 |
| 2.3 | 22 |  | 3 | 2 | 3 | 2 | 3 | 2 | 3 |  | 22.5 |
| 2.4 | 21 |  | 3 | 2 | 3 | 2 | 2 | 3 | 2 |  | 21.25 |
| 2.5 | 20 |  | 3 | 2 | 2 | 3 | 2 | 2 | 2 |  | 20 |
| 2.6 | 19 |  | 2 | 2 | 2 | 3 | 2 | 2 | 2 |  | 18.75 |
| 2.7 | 18 |  | 2 | 2 | 2 | 2 | 2 | 2 | 2 |  | 17.5 |
| 2.8 | 17 |  | 2 | 2 | 2 | 2 | 2 | 2 | 2 |  | 17.5 |
| 2.9 | 16.5 |  | 2 | 2 | 2 | 2 | 2 | 1 | 2 |  | 16.25 |
| 3 | 16 |  | 2 | 2 | 2 | 2 | 2 | 1 | 2 |  | 16.25 |
| 3.1 | 15 |  | 2 | 1 | 2 | 2 | 2 | 1 | 2 |  | 15 |
| 3.2 | 14 |  | 2 | 1 | 2 | 1 | 2 | 1 | 2 |  | 13.75 |
| 3.3 | 13.5 |  | 2 | 1 | 2 | 1 | 2 | 1 | 2 |  | 13.75 |
| 3.4 | 13 |  | 2 | 1 | 2 | 1 | 1 | 2 | 1 |  | 12.5 |
| 3.5 | 12 |  | 2 | 1 | 2 | 1 | 1 | 2 | 1 |  | 12.5 |
| 3.6 | 11.5 |  | 2 | 1 | 1 | 2 | 1 | 1 | 1 |  | 11.25 |
| 3.7 | 11 |  | 2 | 1 | 1 | 2 | 1 | 1 | 1 |  | 11.25 |
| 3.8 | 10.5 |  | 1 | 1 | 1 | 2 | 1 | 1 | 1 |  | 10 |
| 3.9 | 10 |  | 1 | 1 | 1 | 2 | 1 | 1 | 1 |  | 10 |
| 4 | 9 |  | 1 | 1 | 1 | 1 | 1 | 1 | 1 |  | 8.75 |
| 4.1 | 8.5 |  | 1 | 1 | 1 | 1 | 1 | 1 | 1 |  | 8.75 |
| 4.2 | 8 |  | 1 | 1 | 1 | 0 | 1 | 1 | 1 |  | 7.5 |
| 4.3 | 7.5 |  | 1 | 1 | 1 | 0 | 1 | 1 | 1 |  | 7.5 |
| 4.4 | 7 |  | 1 | 1 | 1 | 0 | 1 | 1 | 1 |  | 7.5 |

**FIGURE 1**
